# Principle and Mechanism of Double Eyelid Formation

**DOI:** 10.1055/s-0042-1751100

**Published:** 2023-03-28

**Authors:** Inchang Cho

**Affiliations:** 1 BIO Plastic Surgery Clinic, Seoul, Korea

**Keywords:** double eyelid, curvature, fixation

## Abstract

Some of Asian eyelids have double fold and some do not. Many people tend to prefer double eyelid in aesthetic and functional reasons. Since the mechanism of double eyelid is bonding the eyelid skin to the eye-opening tissue, the principle of double eyelid surgery is also connecting the eyelid skin to levator component. Double eyelids are differentiated by their shape according to the height and curvature. The double eyelid surgery procedure is divided into incision method and nonincision method. And the incision method is subdivided into double fold line design, skin and oculi muscle incision or excision, pretarsal or preaponeurotic soft tissue excision, fixation of posterior lamella to anterior lamella, and skin suture. The nonincision method is to connect the posterior lamella and the anterior lamella as a thread only without an incision. A successful double eyelid surgery creates a fold well-balanced in height, curvature, and depth of the fold based on patient's preference. In this article, the author's own methods of performing surgery are described, with a step-by-step guide and surgical tips.

## Introduction

Double eyelids are not very common for Asian eyes, but many people tend to prefer them. The eyes with double eyelids generally look large, alert, and expressive, while the eyes without them may look heavy and droopy as some of the eyelid skin can cover the eye fissure. People without double eyelids tend to elevate eyebrow using forehead muscles due to the drooping eyelids and can also suffer from entropion. In addition, the eyes without double eyelids may suggest mysterious or introverted, as opposed to the engaging expressiveness of double eyelids. Since the mechanism of the double eyelid is the bonding of the eyelid skin to the eye-opening muscle, the principle of double eyelid surgery is also connecting the eyelid skin to the levator muscle. Double eyelid surgery should make eyes large and pronounced, and the eyelashes exposed, However, an unskilled double eyelid surgery can make eyes look wild and unnatural, even creating the expression of discontentment or disapproval. This article was written to aid surgeons in their practice, presenting the methods the author uses for a satisfactory outcome of the double eyelid procedure.

## Principle

### Why Does the Double Fold Operation Affect the Height of Palpebral Fissure?

The external visibility of an eyeball is determined by the size of palpebral fissure and the amount of eyelid skin drooping over the upper eyelid margin.

There are two controversial factors that affect the height of palpebral fissure. What makes the eyes appear larger or smaller?

### Changes in Palpebral Fissure Size after Double Eyelid Operation

#### What makes Palpebral Fissure Larger?

**Fig. 1:**
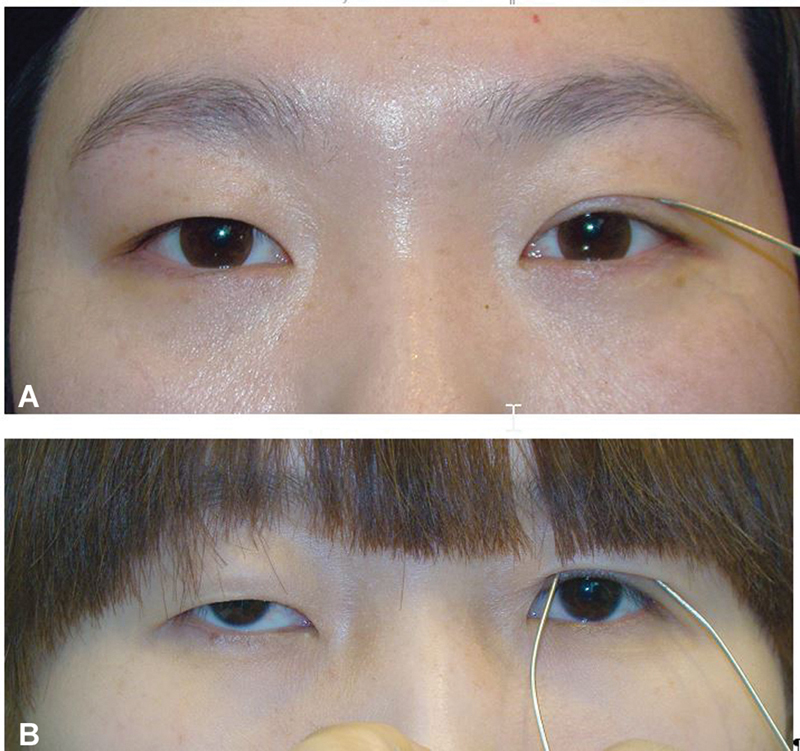
Changes in palpebral fissure with double eyelid formation. Changing amount is different by the skin drooping amount and levator function. ( **A** ) A little eyelid skin drooping. ( **B** ) Much eyelid skin drooping.

- The drooping eyelid skin is elevated ( Fig. 1 ).
- The upper eyelid is lighter from excision of soft tissue.

#### What makes Palpebral Fissure Smaller?

- Double fold fixation leads to increased physical load on the levator muscle.
- After drooping skin elevated, levator and eyebrow compensating effort is disappeared.

## Surgical Procedures

### Design of the Upper Eyelid Crease

The upper eyelid crease should be designed with the patient in the upright sitting position. The height and shape of the crease are determined by various factors. The postoperative shape of the crease can be simulated using an eyelid stylus with the patient in the sitting position and demonstrated to the patient with a mirror. The height of the eyelid crease differs between sitting and supine position and the amount of skin to be excised cannot be evaluated accurately in the supine position. Determining the crease shape is a consultation process that requires conversation with the patient.

#### Types of Eyelid Crease

Double eyelid can be classified by the curvature, height, and depth of the crease.

#### Classification by Curvature

**Fig. 2:**
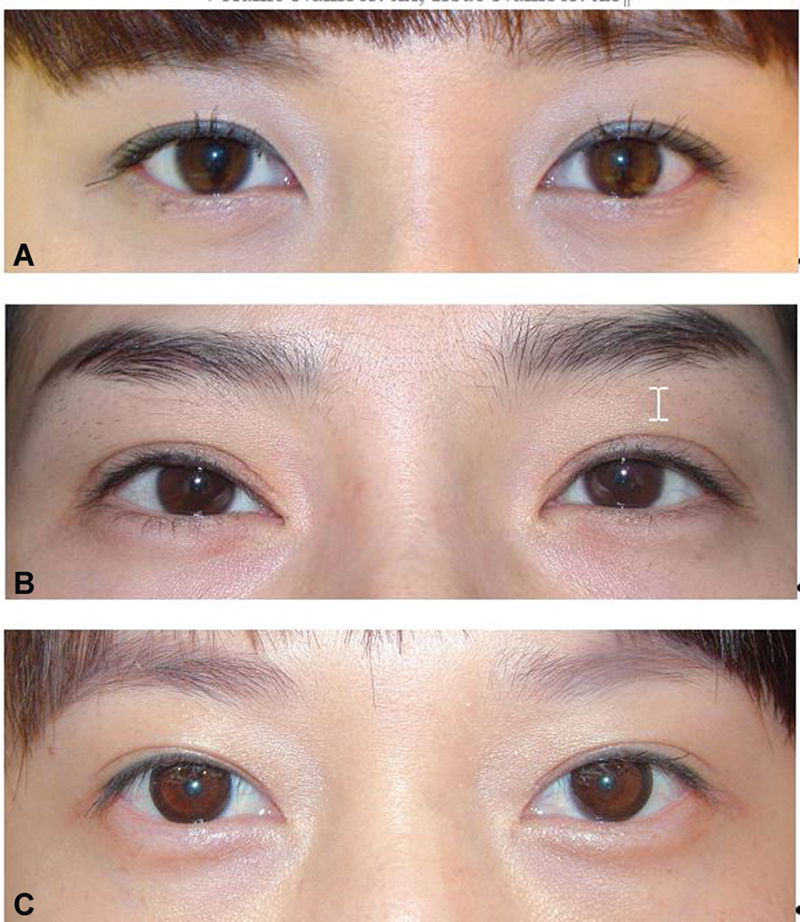
( **A** ) Inside fold. ( **B** ) Outside fold. ( **C** ) Neutral fold (in-out fold).

Upper eyelid crease can be divided as being either inside or outside folds or as being either fan-shaped or parallel. Inside folds are fan shaped, whereas outside folds can be either fan shaped or parallel folds ( Fig. 2 ).

#### The Height of Eyelid Crease

The height of eyelid crease should be selected by temporary stylus with a sufficient amount of discussion. High folds tend to require longer recovery time, whereas lower folds result in in quicker recovery. The biggest problem with high folds is the hypertrophy of pretarsal area, which is described as “sausage eyelid” among Korean patients. One disadvantage of lower fold is that the skin above the crease can sag over the years. On the contrary, the eyelid does not sag as much for higher fold.

The height of the fold is determined by both patient factors and surgical factors. The patient factors are such as skin thickness, skin laxity, the amount of subcutaneous tissue, brow ptosis, levator function, and hollowness of eyelid. And surgical factors are height of the design, depth of the fold, amount of skin excision, and degree of ptosis correction.

## Mechanism behind the Eyelid Crease

The eyelid crease is formed by connecting between anterior and posterior lamella, what we say between orbicularis muscle and tarsal plate or levator aponeurosis. According to this mechanism, an eyelid crease can be created by the surgical formation of an adhesion between anterior lamella and posterior lamella.

**Fig. 3:**
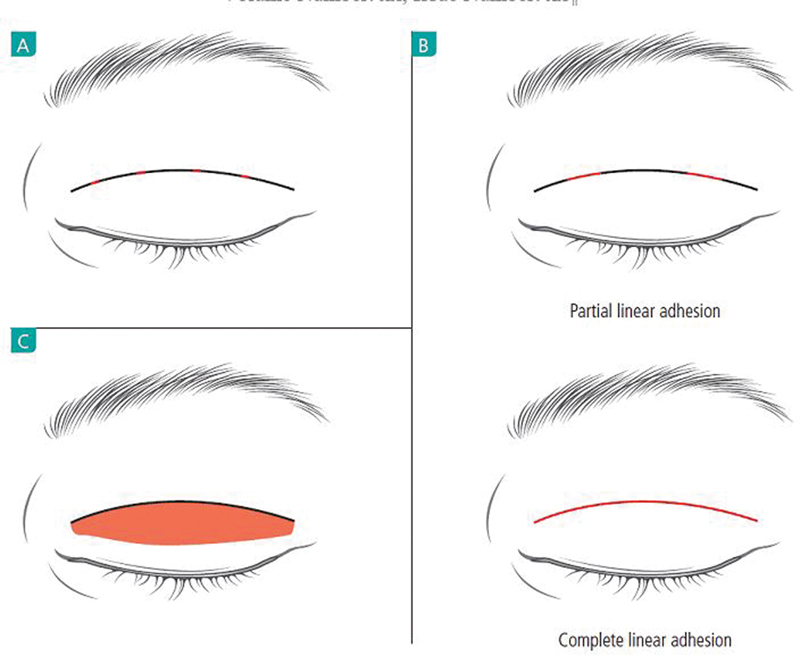
Adhesion types. ( **A** ) Point adhesion. ( **B** ) Linear adhesion. ( **C** ) Planar adhesion. Red lines/area signify adhesion between anterior and posterior lamellae.

The surgically created adhesions can be categorized as point adhesion, linear adhesion, or planar adhesion ( Fig. 3 ). The amount of adhesion is larger for linear adhesion compared with point adhesion, and in planar adhesion compared with linear adhesion. And the chance of loosening of the fold is lowest for planar adhesion and the chance of shallow fold is higher for point adhesion. For this reason, point adhesion is appropriate for creating fold in thinner eyelid.

### Soft Tissue Excision

Linear adhesion is enough for maintaining fold, but planar adhesion makes too much scar tissue.

#### Orbicularis Oculi Muscle

The orbicularis oculi muscle is not excised for nonincision or partial incision method; however, the muscle is excised, when accompanied by large amounts of skin excision in open blepharoplasty. It is important to preserve this muscle near the incision line to minimize the risk of depressed scar.

The author recommends against the undermining of lower flap or the excision of orbicularis oculi muscle on the lower flap. Dissection of the lower flap causes prolonged edema. Another important complication arises from one of the functions of the pretarsal orbicularis muscle, which serves to pump and distribute tear with each blinking movement. As such, excision of this muscle can lead to a significant disability in lacrimal function and the development of dry eye syndrome. The weakness in closing the eyes may contribute to nocturnal lagophthalmos.

#### Pretarsal Soft Tissue

Excision of pretarsal soft tissue promotes the adhesion between the anterior lamella (orbicularis oculi muscle) and posterior lamella (tarsus or levator aponeurosis). This is important if there is an excess of pretarsal fat on the medial tarsus that can cause bulging and increase the likelihood of fixation failure, because of weak adhesion.

#### Orbital Fat and Retro-orbicularis Oculi Fat

In bulky upper eyelid, buttonhole incision is made on the septum to remove orbital fat. Large defect in orbital septum can cause unwanted adhesion that can lead to triple fold.

Also, excessive excision of orbital fat should be avoided unless the upper eyelid is extremely bulky, because the volume of orbital fat decreases with aging. If the excision of the orbital fat is insufficient, the upper eyelid bulk can be further reduced with the excision of retro-orbicularis oculi fat (ROOF). Excision of ROOF in the medial eyelid may cause triple fold and excision of the ROOF should be conservative for the triple fold.

### Depth of Eyelid Crease

Variations in the depth of eyelid crease are shallow crease and deep fold and natural looking fold. Shallow fold looks incomplete. And deep fold has tugging sensation of the eyelid and depressed scar and looks artificial.

#### All Eyelid Creases Become Shallow to a Degree with Time

**Fig. 4:**
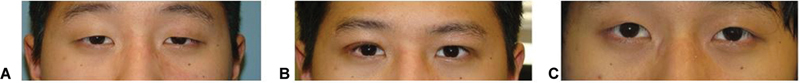
Changes in eyelid fold over time. ( **A** ) Before operation, shallow fold. ( **B** ) After operation, the upper eyelid crease is distinct. ( **C** ) After 5 months, the crease has faded significantly.

The depth of eyelid crease is the most important and difficult problem to be solved. The depth changes with time, surgically created eyelid creases tend to become fainter or becomes undone that makes it much more difficult to communicate the expected outcome of the patient for instance. In eyelids with great resistance against crease formation, operator should make the fold deeper. The crease depth changes vary widely depending on patient factors as well as operative technique factors ( Fig. 4 ).

**Patient factors** : high resistance to crease formation can be expected for the following:

- Thick eyelid skin, younger patient
- Hypertrophic soft tissue
- Severe dermatochalasis
- Ptotic eyelid
- High tension in the medial skin from epicanthal folds
- Past history of failed eyelid crease operation

**Operation technique factors** that make the eyelid crease shallower with time:

- No pretarsal soft tissue excision
- Nonincision technique
- Lower fixation
- Inappropriate fixation method
- Severe edema or hematoma

### Fixation

In the eyelid crease operation, a fixation is defined as a surgically created connection between the anterior lamella (skin and orbicularis oculi muscle) and posterior lamella (tarsus and aponeurosis).

Such a fixation can be considered in the following three characteristics.

- Tissues of fixation
- Location of fixation
- Direction of fixation

#### Tissues of Fixation

##### Fixation of the Anterior Lamella

On the anterior lamella, the author fixated the orbicularis oculi muscle at the edge of lower eyelid flap instead of dermis. 2 Dermis is too thin to be fixed and dermis fixation can lead to depressed scar and inflammation.

##### Fixation of the Posterior Lamella

The tarsal plate and levator aponeurosis are the major fixation tissues in the posterior lamella. The following tissues may be used as method of fixation.

- Tarsus
- Tarsus and attached aponeurosis
- Aponeurosis
- Dual fixation on the tarsus and aponeurosis; Fixation with tarsus and aponeurosis in separate bite to the orbicularis muscle. It is called TAO fixation
- Anterior septum or posterior septum

#### Advantages of TAO Fixation

**Fig. 5:**
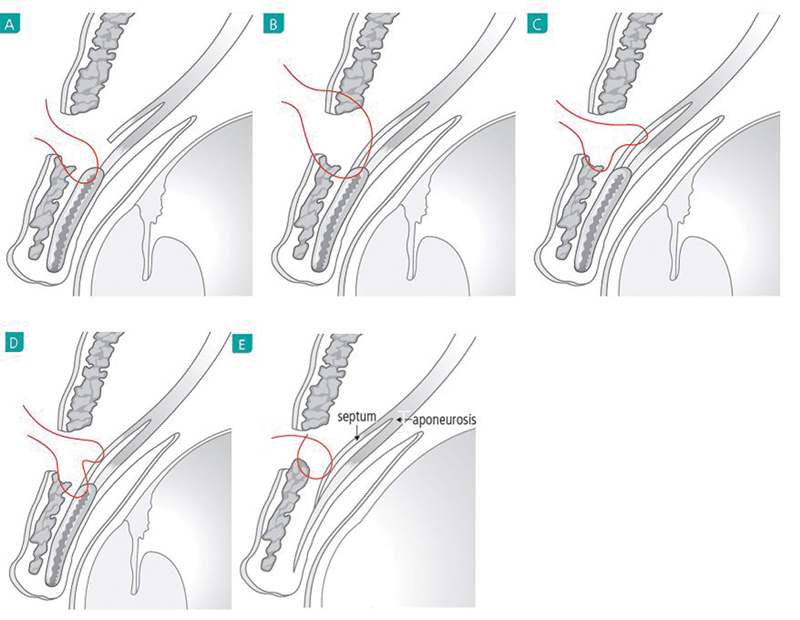
Various fixation methods for the posterior lamella. ( **A** ) Orbicularis muscle to the tarsal plate. ( **B** ) Concomitant fixation to the pretarsal levator aponeurosis and tarsal plate. ( **C** ) Direct fixation to the levator aponeurosis. ( **D** ) Concomitant fixation to the tarsal plate and the levator aponeurosis in separate bite (TAO fixation). ( **E** ) Fixation to the septum or aponeurosis in the lateral portion of eyelid.

- This method avoids the creation of ectopic, deep crease as well as crease loosening.
- The recovery is quick.
- The operation does not decrease the levator function.
- The technique is applicable in a variety of situation ( Fig. 5D ).

#### Location of Fixation

**Fig. 6:**
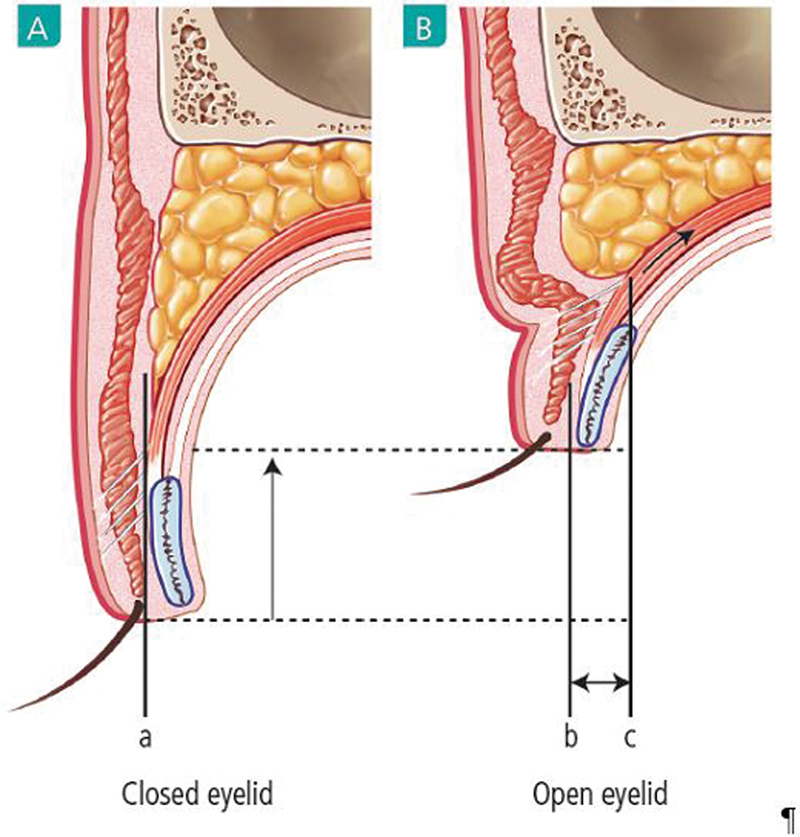
High fixation caused posterior retraction of eyelid crease, which creates a deep fold. ( **A** ) Eyelid closed state ( **B** ) Eyelid open state. The distance between line b and c is the depth of eyelid crease.

The fixation height on the tarsal plate or aponeurosis can change the depth of eyelid crease. A high fixation will stretch the lower flap skin, which will create a deep fold with ectropion ( Fig. 6 ). In contrast, a low fixation creates a shallow crease with the lower flap skin covering the base of eyelashes. To create a crease of moderate depth, the lower flap should be fixed to eyelash slightly everted, which becomes natural over time. In patient with thin skin that is common in elderly patients, the skin should not be fully extended. On the contrary, in patient with thick skin, the skin should be moderately extended for preventing from loosening. The height of fixation should be adjusted according to the factors influencing the eyelid resistance and the type of operation. And compared with the skin incision, the medial portion of crease is fixated at a higher than central or lateral crease.

#### Direction of Fixation

**Fig. 7:**
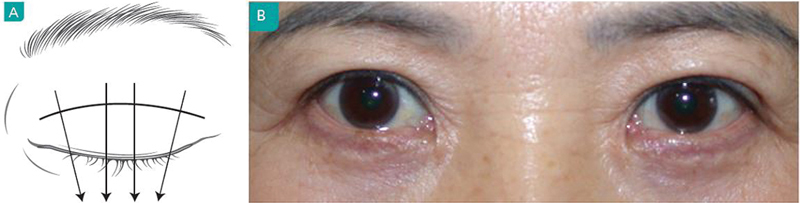
( **A** ) Direction of fixation. The fixation should go from the tarsal plate medially to the orbicularis muscle to compensate for the radial movement of aponeurosis. ( **B** ) The lack of medial compensation can result in the wrinkles observed superior to the double fold.

Fixation is performed in a radial direction ( Fig. 7 ). Medially, the orbicularis muscle should be fixed to the tarsal plate more medially to the orbicularis muscle. If not, it may result in the wrinkles observed superior to the double fold.

### Length of Eyelid Crease

**Fig. 8:**
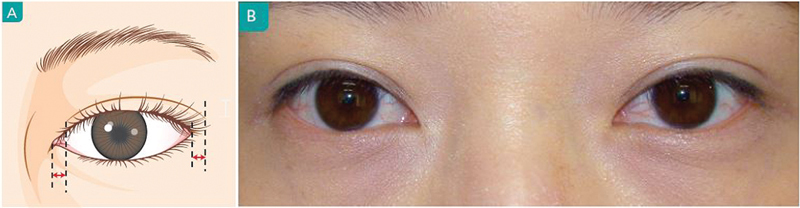
( **A, B** ) Length of eyelid crease.

Supratarsal crease begins 2 to 3 mm away from the medial canthus and extended 4 to 6 mm beyond the lateral canthus ( Fig. 8 ).

Ideally, the crease begins 2 to 3 mm from the medial canthus and extends 4 to 6 mm past the lateral canthus.

### Skin Suture

The edges of the skin incision should be approximated and closed in an everted manner. To avoid a gap in the orbicularis muscles between the upper and lower flap, the skin should incorporate the orbicularis muscle.

## Discussion

There are many different ways of creating double eyelids or the supratarsal crease, and the above-mentioned procedures are the author's preferred method. Regardless of the procedure, the depth of the fold is the most important yet most difficult factor to control. The depth changes and becomes shallower over time. The crease depth can vary widely over time depending on the patient as well as operational variations, which makes it very difficult to communicate the expected outcome to the patient. There are varying levels of resistance to the eyelid crease, and eyelids with higher resistance require a deeper crease. The amount of resistance to the eyelid crease can be observed by simulating the crease in the sitting position. Only a light touch of the stylus is required to simulate an eyelid crease for low-resistant eyelids, and the temporary crease tends to remain in place for a while. In contrast, the disappearance of the crease as soon as the stylus is removed indicates higher resistance that requires a deeper crease. The height of fixation should be at the point where the lower flap skin is stretched 80 to 90% of its full length for thin eyelid skin, and same as the full length for thick eyelid skin.

## Conclusion

Some of Asian eyelids have double fold and some do not. Eyelids without double fold are more common for Asians than with double fold, but many people prefer double fold for both aesthetic and functional reasons. There is no single best approach to the double eyelid surgery because the desired fold shape is different depending upon several factors including race, culture, age, and individual characteristics. The shapes are determined by the fold height, depth, and curvature. For desired outcome, we need to thoroughly understand the eyelid anatomy, the mechanism of a double fold, and apply various surgical options as needed.
